# Ferroptosis-related gene signature predicts the prognosis in Oral squamous cell carcinoma patients

**DOI:** 10.1186/s12885-021-08478-0

**Published:** 2021-07-20

**Authors:** Hongyu Li, Xiliu Zhang, Chen Yi, Yi He, Xun Chen, Wei Zhao, Dongsheng Yu

**Affiliations:** 1 Hospital of Stomatology, Guanghua School of Stomatology, Sun Yat-sen University, Guangzhou, 510030 Guangdong China; 2 Guangdong Provincial Key Laboratory of Stomatology, Guangzhou 510030 Guangdong, China; 3 Department of Pediatric Dentistry, Hospital of Stomatology, Guanghua School of Stomatology, Sun Yat-sen University, Guangzhou, 510030 Guangdong China; 4 Department of Oral Emergency, Hospital of Stomatology, Guanghua School of Stomatology, Sun Yat-sen University, Guangzhou, 510030 Guangdong China

**Keywords:** Oral squamous cell carcinoma, Ferroptosis, Gene prognosis signature, Nomogram, Immunity

## Abstract

**Background:**

The prognosis of oral squamous cell carcinoma (OSCC) patients is difficult to predict or describe due to its high-level heterogeneity and complex aetiologic factors. Ferroptosis is a novel form of iron-dependent cell death that is closely related to tumour growth and progression. This study aims to clarify the predictive value of ferroptosis-related genes (FRGs) on the overall survival(OS) of OSCC patients.

**Methods:**

The mRNA expression profile of FRGs and clinical information of patients with OSCC were collected from the TCGA database. Candidate differentially expressed ferroptosis-related genes (DE-FRGs) were identified by analysing differences between OSCC and adjacent normal tissues. A gene signature of prognosis-related DE-FRGs was established by univariate Cox analysis and LASSO analysis in the training set. Patients were then divided into high- and low-risk groups according to the cut-off value of risk scores, A nomogram was constructed to quantify the contributions of gene signature and clinical parameters to OS. Then several bioinformatics analyses were used to verify the reliability and accuracy of the model in the validation set. Finally, single-sample gene set enrichment analysis (ssGSEA) was also performed to reveal the underlying differences in immune status between different risk groups.

**Results:**

A prognostic model was constructed based on 10 ferroptosis-related genes. Patients in high-risk group had a significantly worse OS (*p* < 0.001). The gene signature was verified as an independent predictor for the OS of OSCC patients (HR > 1, p < 0.001). The receiver operating characteristic curve displayed the favour predictive performance of the risk model. The prediction nomogram successfully quantified each indicator’s contribution to survival and the concordance index and calibration plots showed its superior predictive capacity. Finally, ssGSEA preliminarily indicated that the poor prognosis in the high-risk group might result from the dysregulation of immune status.

**Conclusion:**

This study established a 10-ferroptosis-releated gene signature and nomogram that can be used to predict the prognosis of OSCC patients, which provides new insight for future anticancer therapies based on potential FRG targets.

**Supplementary Information:**

The online version contains supplementary material available at 10.1186/s12885-021-08478-0.

## Background

Oral squamous cell carcinoma (OSCC) is one of the most common head and neck malignancies [1, 2]. There are more than 300,000 new cases each year, accounting for approximately 2 to 3% of all cancers worldwide [2]. Alcohol abuse, smoking, areca nut chewing and chronic HPV or HBV infections are considered high-risk factors for OSCC [3–7]. The five-year overall survival (OS) rate for OSCC remains at approximately 40–50% despite recent advances in diagnostic and therapeutic approaches [8]. It is difficult to predict or describe the prognosis of OSCC because of its high-level heterogeneity and complex aetiologic factors. Most of the current clinical prediction models have been developed based on histological characteristics, which involves the risk of tissue trauma and tumour irritation. Therefore, it is essential to explore a new and noninvasive prognostic model from the perspective of the biological behaviour of tumour cells to facilitate early detection and optimize treatment strategies for OSCC.

Ferroptosis is a recently discovered type of regulated cell death (RCD) that differs morphologically, biochemically, and genetically from apoptosis, autophagy, and necrosis and is characterized by iron-dependent and lethal reactive oxygen species (ROS) and lipid peroxidation(LPO) accumulation [9, 10]. In recent years, inducing ferroptosis in cancer cells become a latent target for cancer therapy [11–13], especially in those drug-tolerant and radiation resistant cancers [14, 15]. Previous studies have also demonstrated that ferroptosis plays important roles in OSCC. Several drugs (such as telaglenastat (CB-839) [16] and histone deacetylase inhibitor quisinostat [17]) and new materials such as zero-valent iron nanoparticles [18] have been shown to enhance the anti-OSCC response in part through ferroptosis. Moreover, both non-thermal plasma and photodynamic therapy(PDT) can effectively eliminate OSCC cells by inducing ferroptosis [18–20]. Some genes, such as GPX4 and SREBP, that promote OSCC cell proliferation appeared to protect cells from ferroptosis [21]. Zhu’s report [19] also showed that the ferroptosis-negative regulatory gene SLC7A11 was upregulated in OSCC, and their team overcame hypoxia-associated resistance to PDT by activating ferroptosis in OSCC cells. Although some studies have employed ferroptosis-related genes to construct survival prognostic models for some cancers [22–24], the predictive value of ferroptosis-related genes on the overall survival of OSCC patients warrants further and more detailed studies.

**Fig. 1:**
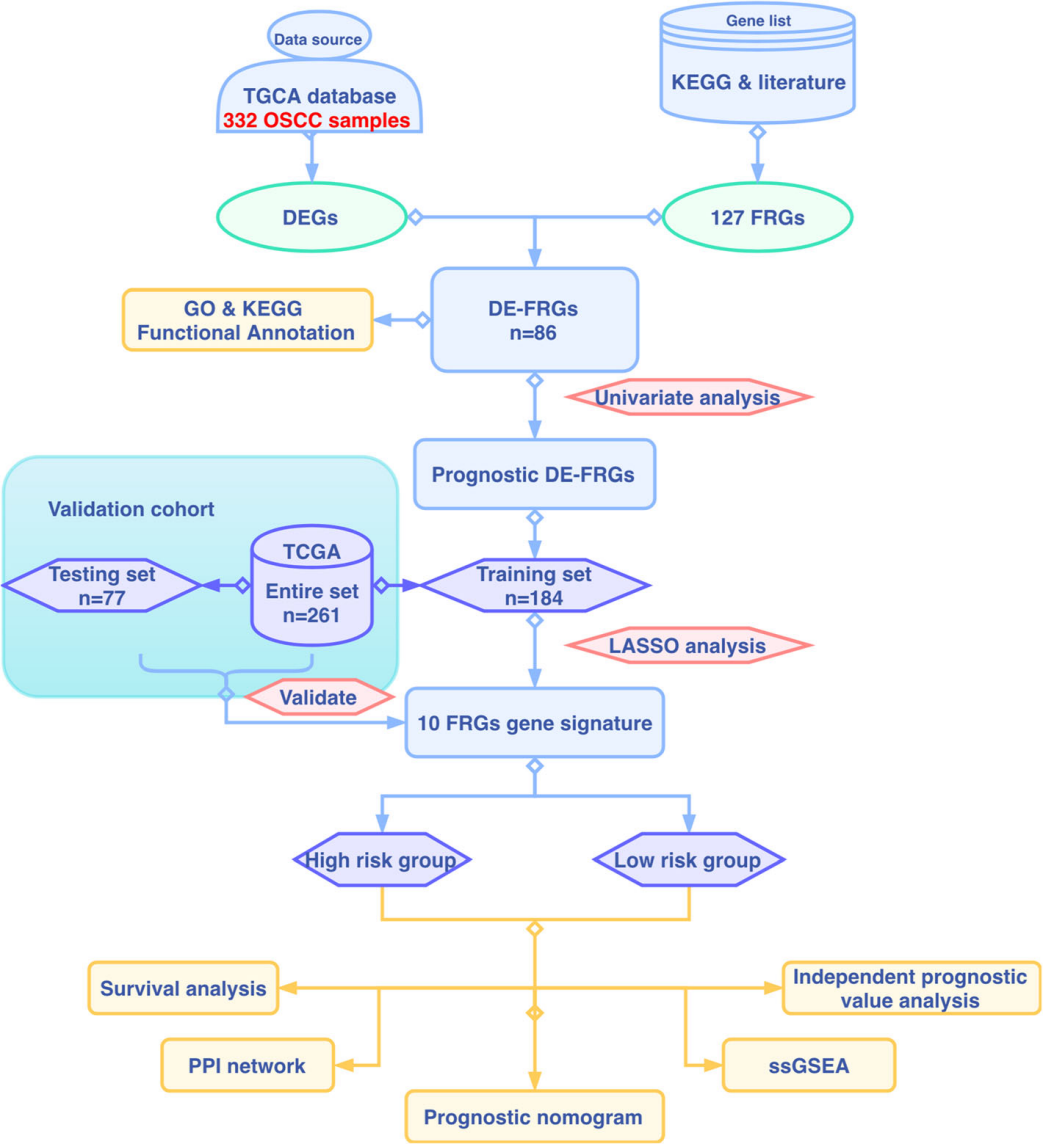
Flowchart of the construction and validation of the ferroptosis-related gene (FRG) signature and nomogram. TCGA, The Cancer Genome Atlas; OSCC, oral squamous cell carcinoma; DEGs, differentially expressed genes; DE-FRGs, differentially expressed ferroptosis-related genes; LASSO, least absolute shrinkage and selection operator; GO, Gene Ontology; KEGG, Kyoto Encyclopedia of Genes and Genomes; ssGSEA, single-sample gene set enrichment analysis; PPI, protein-protein interaction

In this study, we explored the promising value of ferroptosis-related genes as biomarkers in targeted therapy and their prognostic value for the survival rate of OSCC. We downloaded the gene expression profile and clinical information of OSCC from The Cancer Genome of Atlas (TCGA). Then, a FRG-based prognostic signature and nomogram were constructed in a training set and verified in a validation set. Finally, single-sample gene set enrichment analysis (ssGSEA) was applied to reveal the latent mechanism of correlation between immune status and the risk model. The flow chart of the present study is shown in Fig. 1.

## Methods

### Data source

The mRNA expression data of 127 ferroptosis-related genes and corresponding clinical data of OSCC patients were retrieved from The Cancer Genome Atlas official website (TCGA, https://portal.gdc.cancer.gov/), including 306 tumour samples and 30 normal samples. The present study followed the TCGA Data Access Policy and Publication Guidelines. The list of 127 FRGs was retrieved from Kyoto Encyclopedia of Genes and Genomes (KEGG) and previous literature. Detailed information on these FRGs is summarized in Additional file 1: Table S1.

### Identification of differentially expressed ferroptosis-related genes (DE-FRGs)

The EdgeR package in R software (Vision 3.5.2) was utilized to normalize the RNA-sequencing data and perform differential analysis between OSCC and adjacent normal tissues. FRGs with a false discovery rate (FDR) of less than 0.05 were considered as DE-FRGs. Next, DE-FRGs were displayed in a heat map, volcano plot and boxplot by the ggplot2 package.

### Functional annotation of DE-FRGs

Gene Ontology (GO) and KEGG analysis were performed using clusterprofile package to explore the potential function of the DE-FRGs and the results were plotted by ggplot2 package. Results were considered significantly different if *p* < 0.05.

### Construction of prognostic signature based on FRGs

OSCC patients with a follow-up time of less than 30 days were excluded and only 296 tumour samples were included for further analysis. Univariate Cox analysis was used to select prognosis-related FRGs. Given that the small number of ferroptosis-related genes, a strict cut-off value may result in a loss of genes with prognostic potential. Therefore, *p* < 0.1 was applied as the cut-off value in univariate Cox analysis. Candidate prognosis-related DE-FRGs were determined after overlapping the DE-FRGs and the prognosis-related FRGs. The hazard ratio (HR) and the 95% confidence interval (CI) of DE-FRGs were represented by a forest map.

There were 261 patients who provided complete information on survival and important clinical parameters. Patients with integrated clinical information were randomly and homogeneously split into a training set (184 OSCC patients) and a testing set (77 OSCC patients). Least absolute shrinkage and selection operator (LASSO) analysis was next performed to develop a multigene prognostic risk model with candidate prognosis-related DE-FRGs in the training set based on the optimal value of lambda (λ). The glmnet package was used to implement the procedures mentioned above. To present the interactions between these prognostic genes, a protein-protein interaction (PPI) network of 10 FRGs and an additional 20 related-genes was constructed in STRING (https://string-db.org/), and Cytoscape (Version 3.8.0) was used for visualization.

A FRG-based risk score for each sample was calculated by the following formula: Risk score = Gene exp1 × β1 + Gene exp2 × β2 + … + Gene exp. n × βn (Gene exp. indicates the value of gene expression while β stands for the corresponding LASSO regression coefficient). The survival ROC package was used to perform time-dependent receiver operating characteristic (ROC) analysis to determine the optimal cut-off value of the risk score, and then the cut-off value stratified patients into high and low-risk groups. Kaplan-Meier (K-M) survival curve analysis was employed to analyse OS and compared by log-rank test. The area under the ROC curve (AUC) was used to evaluate the predictive sensitivity and accuracy of the prognostic model.

### Validation of the FRG-based signature in OSCC

To confirm the prognostic capability of the FRG-based signature model, the risk score was calculated with the consistent risk score formula in the testing set and entire set and patients were stratified with the identical optimal cut-off value. K-M survival curve analysis and ROC analysis were performed as aforementioned.

### Independent prognostic value analysis

Univariate analysis was used to evaluate each predictive value of the risk model and clinical parameters (age, gender, TNM stages, smoking and alcohol) while multivariate Cox analysis for OS was employed to confirm the independent risk variables. The correlation between risk score and the clinical characteristics was further examined by Pearson’s correlation analysis.

### Construction and validation of a predictive nomogram

To predict the overall survival rate for OSCC, a nomogram with FRG-based risk score and clinical risk factors was created by using the rms package. The calibration curves graphically represent the consistency between actual and predicted OS for OSCC individuals by using this nomogram. The concordance index (C-index) ranging from 0.5–1.0 (0.5 indicates a random chance while 1.0 indicates a remarkable ability) was also used to determine the predictive accuracy of the nomogram. Finally, the predictive performance of the nomogram was confirmed in the testing set and the entire set.

### Single-sample gene set enrichment analysis (ssGSEA)

ssGSEA was applied to determine the signaling pathways with different enrichment scores between subgroups. The enrichment score of each sample was calculated with the gsva package. The limma package was then implemented to identify enriched gene sets that were significantly different between groups and showed the top 50 gene sets by heatmap. Correlations between the risk score and these enriched pathways were determined by performing Pearson’s correlation analysis.

### Immune cell infiltration patterns between subgroups

To clarify the possible correlations between the immune status and FRG-based prognostic signature. ssGSEA was applied to identify the immune cell infiltration patterns and calculate the enrichment scores of 16 immune cells and 13 related functions.

### Statistical analysis

All analyses were performed with R software (Version 3.5.2) and Microsoft Excel (Version 16.38). If not specifically mentioned, *p* < 0.05 was considered statistically significant or taken as the cut-off criterion.

## Results

### Identification of differentially expressed ferroptosis-related genes (DE-FRGs) in OSCC

**Fig. 2:**
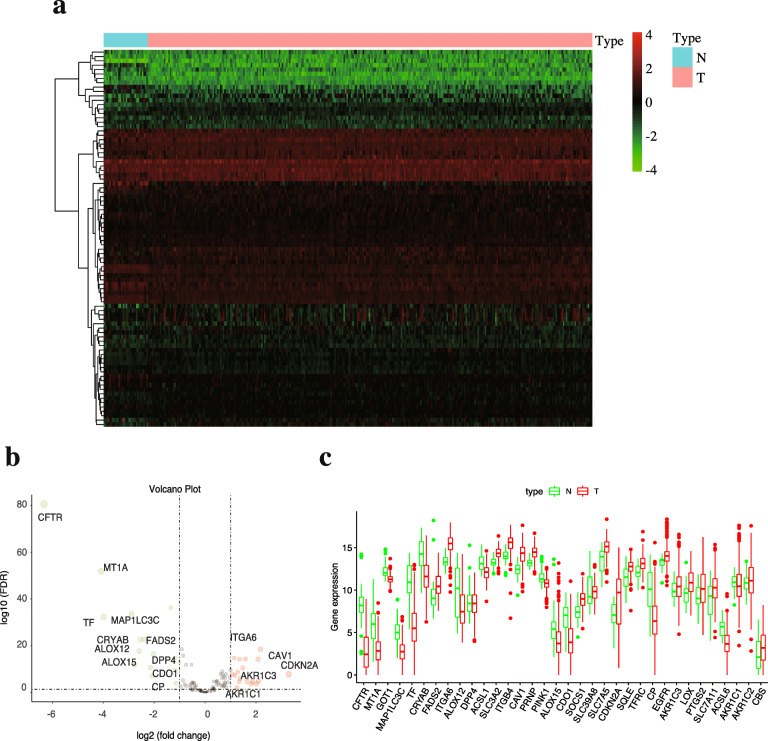
Identification of differentially expressed ferroptosis-related genes (DE-FRGs) based on TCGA database. **(a)** Heatmap and **(b)** Volcano plot of 86 DE-FRGs between normal and OSCC tissues. The changing colour from green to red indicates the low-to-high level of gene expression in the heatmap. The Volcano plot also lists the names of the genes with |log_2_(FC) | > 2. **(c)** Scatter plot shows the difference in the expression of 34 DE-FRGs (with the criterion of FDR < 0.05 and|log_2_(FC)| > 1) in OSCC (red) and normal (green) tissue. N, normal tissue. T, OSCC tissue

A total of 86 FRGs expressed differentially between OSCC and normal tissue (FDR < 0.05), were called DE-FRGs, among which 36 genes were downregulated and 50 were upregulated. The DE-FRGs are displayed in a heatmap (Fig. 2a) and volcano plot (Fig. 2b). However, only 34 genes were more significantly differentially expressed when the criteria were set as FDR < 0.05 and |log_2_FC| > 1 (Fig. 2c).

### Functional enrichment analysis of DE-FRGs

**Fig. 3:**
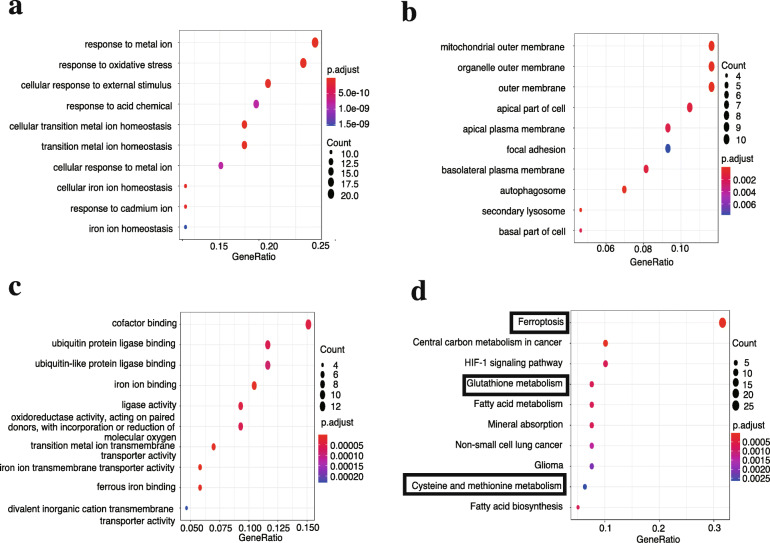
Gene functional annotation of 86 DE-FRGs. GO enrichment analysis (A-C) is presented in three parts: **(a)** biological process, **(b)** cellular component, and (**c)** molecular function. **(d)** KEGG pathway enrichment analysis shows the primary pathways enriched by the 86 DE-FRGs. Three crucial ferroptosis-related signaling pathways are marked by the black box

GO analysis indicated that 86 DE-FRGs were primarily enriched in “response to metal ion”, “response to oxidative stress” and “cellular response to external stimulus” in biological process (BP) (Fig. 3a), “mitochondrial outer membrane”, “organelle outer membrane”, and “autophagosome” in cellular component (CC) (Fig. 3b), and “cofactor binding”, “iron ion binding” and “ubiquitin protein ligase binding” in molecular function (MF) (Fig. 3c). KEGG pathway enrichment analysis suggested that these DE-FRGs were primarily involved in “ferroptosis”, “central carbon metabolism in cancer”, “HIF-1 signaling pathway”, “glutathione metabolism” and “fatty acid metabolism”(Fig. 3d). Both GO and KEGG analysis indicated that these DE-FRGs were closely associated with ferroptosis rather than other types of cell death.

### Determination of the prognostic DE-FRG

**Fig. 4:**
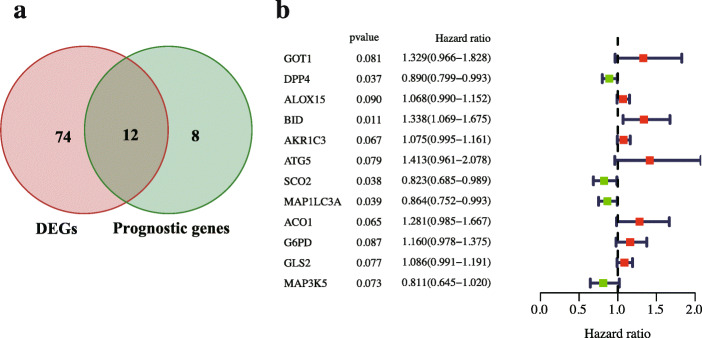
Identification of DE-FRGs with prognostic value. **(a)** Venn diagram shows that 12 genes were screened out the prognosis-related DE-FRGs (*p* < 0.1). **(b)** Forest plots exhibit the hazard ratio and 95% confidence intervals of 12 prognosis-related DE-FRGs by univariate Cox regression analysis

Univariate Cox regression analysis screened 20 FRGs with prognostic value. After overlapping 86 DE-FRGs and 20 prognostic-FRGs, 12 prognostic DE-FRGs were acquired (Fig. 4a). As show in Fig. 4b, most prognostic-related genes were shown as risk genes (HR > 1) in the plot.

### Construction of the FRG-based prognostic signature in OSCC

**Fig. 5:**
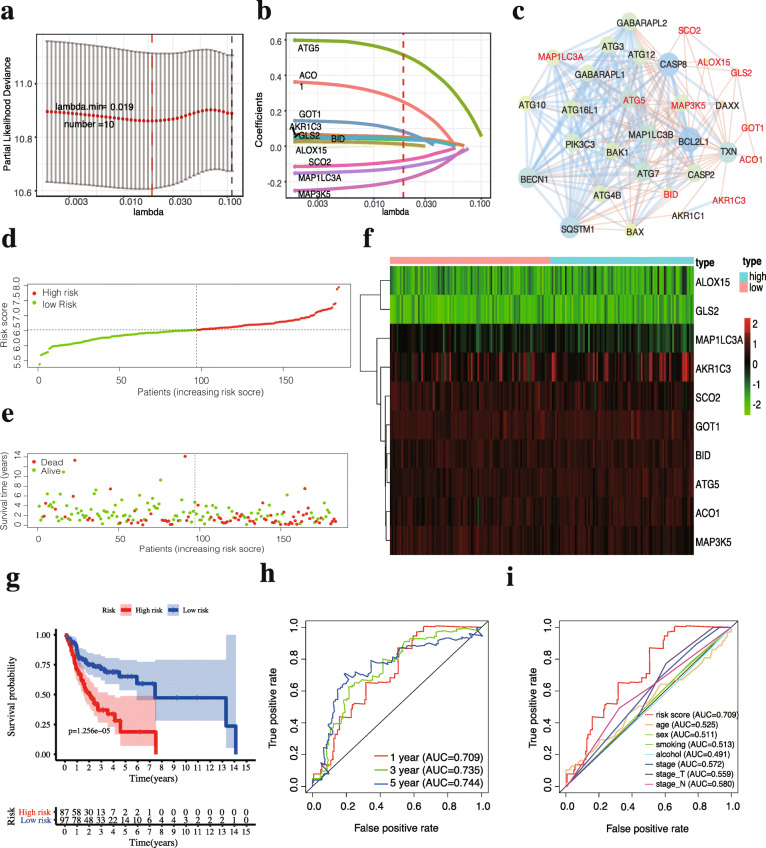
Construction of 10 ferroptosis-related gene signature in the TCGA training set. **(a)** The optimal value of penalty lambda (λ) is selected in LASSO analysis. **(b)** LASSO coefficient profiles of 10 FRGs in OSCC. **(c)** The protein-protein interaction network among 10 candidate prognostic genes (red font) and 20 additional genes. The size of the node and the thickness of the connection line represent the interaction degree and the number of supporting pieces of evidence, respectively. The distribution of risk score **(d)** and survival time and status **(e)**. The optimum cut-off value of the risk score is shown in **(d)** and used to divide each patient into different risk groups. **(f)** Heatmap of mRNA expression of the 10-prognostic FRGs. **(g)** K-M curves for the overall survival between low and high-risk OSCC patients. **(h)** Time-dependent ROC curves for the prognostic model for 1-, 3-, and 5-year OS of OSCC in the training set. **(i)** Time-dependent ROC curves for the risk score and clinical parameters in 1-year OS of OSCC in the training set. The AUC values represent the predictive performance of the gene signature and clinical risk factor

A total of 261 patients with integrated and vital clinical parameters were enrolled in the subsequent model construction (Table 1). LASSO regression analysis determined 10-prognostic-DE-FRGs with the most powerful prognostic values for OSCC in the training set (Fig. 5a-b). Table 2 showed the corresponding regression coefficients and HRs of the 10 prognostic-FRGs. Among these FRGs, seven genes (ATG5, BID, ACO1, GOT1, AKR1C3, GLS2, ALOX15) were regarded as risk genes (HR > 1), and the other three genes (SCO2, MAP1LC3A, MAP3K5) were considered protective genes (HR < 1). Furthermore, the PPI network showed that ATG5, MAP1LC3A and MAP3K5 were core genes among the prognostic genes (Fig. 5c).

**Table 1:** Clinical Characteristics of OSCC patients in the TCGA cohort

Variates	Training set ***n*** = 184	Testing set ***n*** = 77	Entire set ***n*** = 261
**Age**			
< 60	76	35	111
≥ 60	108	42	150
**Gender**			
Male	127	53	180
Female	57	24	81
**TNM stage**			
Stage I-II	35	19	54
Stage III-IV	149	58	207
**T (Tumour size)**			
T1-T2	66	36	102
T3-T4	118	41	159
**N (Lymph Node)**			
N0	85	28	113
N1-N3	99	49	148
**M (Tumour Metastasis)**			
M0	184	77	261
M1	0	0	0
**Smoking history**			
No	44	27	71
Yes	140	50	190
**Alcohol history**			
No	62	23	85
Yes	122	54	176

**Table 2:** Ten OSCC-prognostic ferroptosis-related genes were identified LASSO regression analysis

Gene	HR (95%CI)	LASSO Coefficient
ATG5	1.8 (1.1–2.9)	0.516
BID	1.3 (0.95–1.7)	0.0375
ACO1	1.3 (0.87–1.8)	0.250
GOT1	1.2 (0.78–1.8)	0.0825
AKR1C3	1.1 (0.97–1.2)	0.0504
GLS2	1.1 (0.95–1.2)	0.0421
ALOX15	1.0 (0.94–1.1)	0.0118
SCO2	0.88 (0.69–1.1)	−0.0805
MAP1LC3A	0.85 (0.71–1.0)	−0.119
MAP3K5	0.77 (0.57–1.1)	− 0.188

Next, an individualized risk score was calculated with the formula for the FRG signature: Risk score = expression value of ATG5 * 0.516 + BID * 0.0375 + ACO1 * 0.250 + GOT1 * 0.0825 + AKR1C3 * 0.0504 + GLS2 * 0.0421 + ALOX15 * 0.0118 - SCO2 * 0.0805 - MAP1LC3A * 0.119 - MAP3K5 * 0.188. ROC analysis then confirmed the optimal cut-off value (6.5285) of this prognostic signature and patients were divided into two groups with high (*n* = 87) and low-risk (*n* = 97) according to the optimal value. The distribution of risk score (Fig. 5d), survival status (Fig. 5e) and the expression profile (Fig. 5f) indicated that patients with low-risk had a higher possibility of surviving. K-M analysis also suggested that high-risk group had a significantly poorer prognosis than the low-risk group (Fig. 5g, log-rank *p* = 1.256*10^− 5^). Furthermore, AUC values for 1-year, 3-year, and 5-year OS were 0.709, 0.735 and 0.744, respectively, thereby indicating that the risk model can accurately predict the OS of OSCC patients (Fig. 5h). The ROC curve involving several clinical risk factors and the risk score for 1-year OS showed that this gene signature (AUC = 0.709) had better prediction performance than the other clinical parameters (Fig. 5i).

### Validation of the FRG-based signature in OSCC

**Fig. 6:**
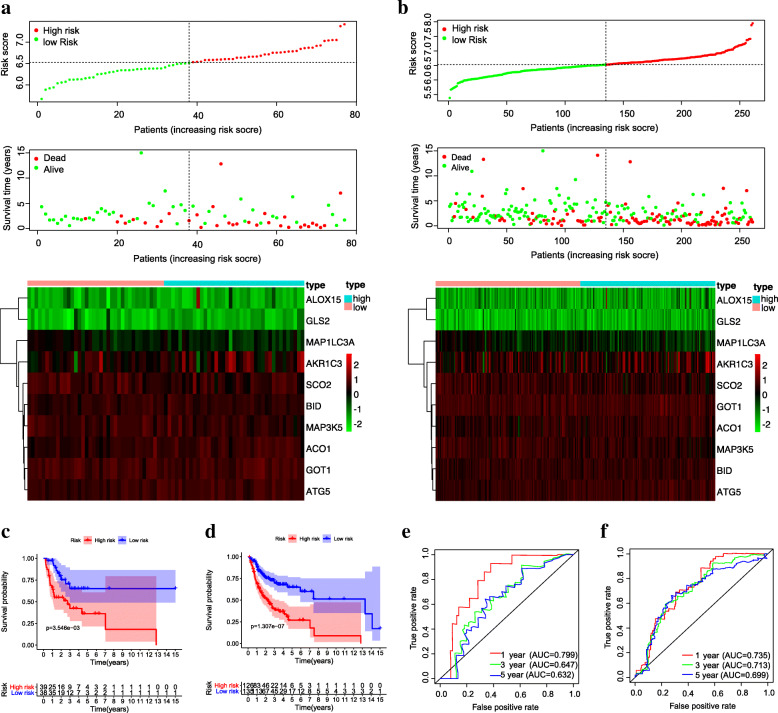
Validation of the 10 FRG signatures in the TCGA testing set and entire set. The distribution of risk score, survival time, OS status and heatmap of 10-FRG gene expression in the testing set **(a)** and entire set **(b).** K-M curves for overall survival of OSCC patients in different subgroups and time-dependent ROC curves in the testing set **(c, e)** and entire set **(d, f)**

To confirm the prognostic capability of the FRG-based signature model, the risk score in the testing set and entire set was calculated with the consistent a formula and patients were classified into two groups with the same cut-off value (6.5285). The distribution of each patient’s risk scores, survival status and expression profile of 10 FRGs in the testing set (Fig. 6a) and entire set (Fig. 6b) showed similar results to the training set, in which patients in the high-risk group had a worse prognosis. K-M survival analysis in both the testing set (Fig. 6c, log-rank *p* = 3.546*10^− 3^) and entire set (Fig. 6d, log-rank *p* = 1.307*10^− 7^) also confirmed that the high-risk groups had a tendency to died earlier. In addition, the AUC values for 1-year, 3-year, and 5-year were 0.799, 0.647 and 0.632 in the testing set (Fig. 6e), and 0.735, 0.713 and 0.699 in the entire set (Fig. 6f), respectively. In summary, all of these results suggested that the 10-FRG gene signature was a credible predictor of overall survival in patients with OSCC.

### Independent prognostic value analysis

**Fig. 7:**
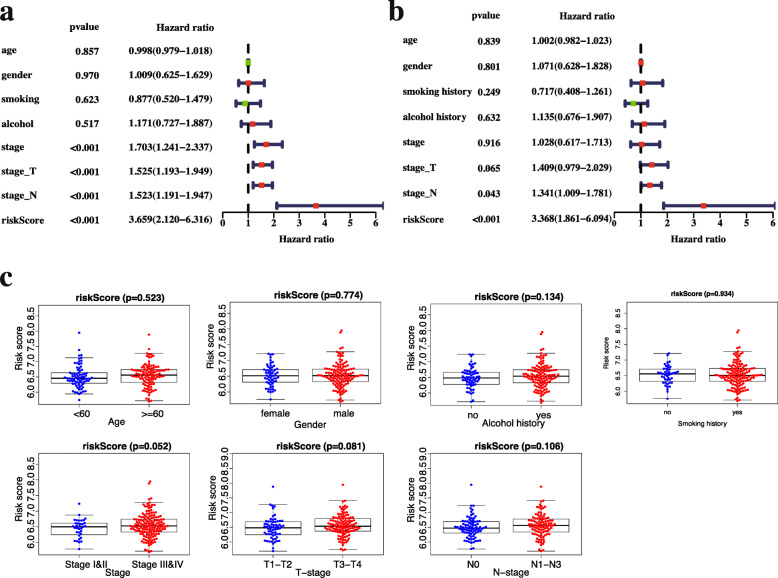
Relationship between risk score and clinical characteristics. The results of univariate **(a)** and multivariate Cox regression analysis **(b)** show the correlation between risk score of the novel prognostic model and clinical characteristics (including age, gender (sex), smoking history (smoking), alcohol history (alcohol), stage, tumour size (stage-T), tumour node (stage-N)) and the overall survival of OSCC patients in the training set. **(c)** Relationship between risk score and age, gender, smoking history, alcohol history, stage, T-stage, and N-stage

Next, univariate Cox analysis indicated that the risk score (HR = 3.659, 95% CI = 2.120–6.316, *p* < 0.001), stage (HR = 1.703, 95% CI = 1.241–2.337, *p* < 0.001), T-stage (HR = 1.525, 95% CI = 1.193–1.949, *p* < 0.001) and N-stage (HR = 1.523, 95% CI = 1.191–1.947, *p* < 0.001) were significantly associated with OS (Fig. 7a). After adjusting for additional clinical features, the multivariate Cox analysis confirmed that the FRG gene signature was an independent predictor for the survival of OSCC patients (Fig. 7b, risk score: HR = 3.368, 95% CI = 1.861–6.094, *p* < 0.001). Additionally, the correlation between risk models and clinical characteristics was evaluated. Unexpectly, none of the clinical factors were significantly related to the risk score (*p >* 0.05). The risk score indicated a possible trend towards significantly higher TNM-stage III-IV (*p* = 0.052), tumour size stages T3-T4 (*p* = 0.081) and lymph node metastasis stages N1-N3 (*p* = 0.106) (Fig. 7c).

### Construction and validation of a predictive nomogram

**Fig. 8:**
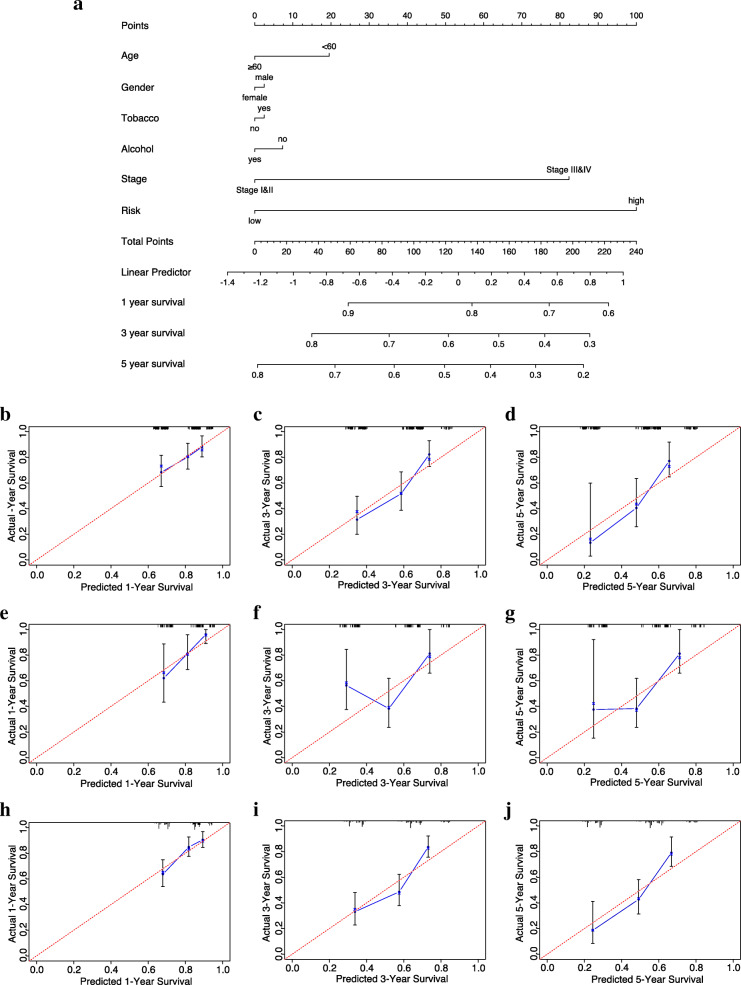
Construction and validation of nomogram. **(a)** The prediction nomogram integrating the risk score and multiple clinical risk factors to predict 1-, 3-, or 5-year survival in OSCC patients. Each factor corresponds to a point value at the top row, indicating their contribution to the OS of OSCC patients. These values were summed to obtain a total point. Then, a line was drawn from the location of “total points” downward to the survival axis to determine the possibility of 1-, 3-, or 5-year survival. **(b-j)** Calibration curves show that the predictive survival fit actual survival well at 1-year **(b)**, 3-years **(c)**, and 5-years **(d)** in the training set. Similar results are obtained with the testing set **(e-g)** and entire set **(h-j)**

Following that, a nomogram was developed on the training set to quantitatively predict patient OS with the FRG signature and some important clinical parameters (Fig. 8a). Points in the nomogram were assigned to represent the contribution of each factor to OS. Not surprisingly, the risk score was the vital OS predictor for OSCC individuals because high-risk accounted for 100 points. Calibration curves in the training set showed that the predictive survival matched the actual survival well at 1-year (Fig. 8b), 3-years (Fig. 8c), and 5-years (Fig. 8d). Plots of the testing set (Fig. 8e-g) and entire set (Fig. 8h-j) also indicated superior agreement in general. In addition, the concordance index (C-index) ranging from 0.5–1.0 (0.5 indicates random chance while 1.0 indicates remarkable ability) was also used to determine the predictive accuracy of the nomogram. The C-index of the nomogram was 0.64 (95% CI = 0.58–0.70, *p* = 3.38*10^− 5^) in the training set, while 0.68 (95% CI =0.58–0.78, *p* = 2.77 × 10^− 4^) in the testing set, and 0.65(95% CI =0.60–0.70, *p* = 3.01 × 10^− 8^) in the entire set, indicating good accuracy and sensitivity of this nomogram. Overall, both calibration curves and the C-index confirmed that the nomogram was a favourable and reliable tool for prognosis prediction in OSCC patients.

### Signaling pathways enrichment analysis between two risk groups

**Fig. 9:**
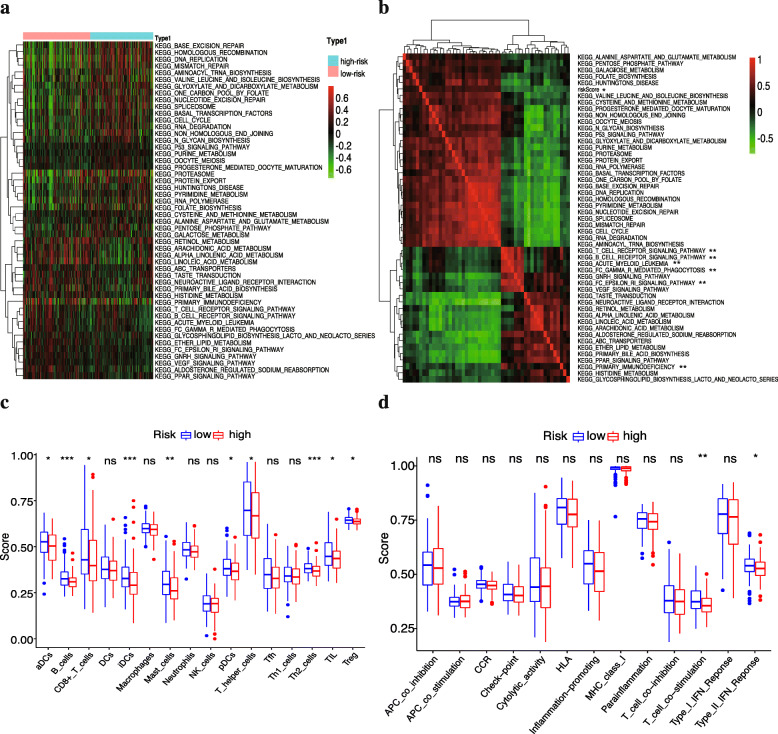
Pathway profiles and single-sample gene-set enrichment analysis (ssGSEA) between the low and high-risk groups across the entire TCGA cohort. **(a)** Enriched pathway profiles of the entire set show the DEGs between different risk scores enriched in different pathways. ssGSEA was performed to calculate the enrichment scores of pathway activity. Each score is displayed in a cell, with a colour change indicating a low (green) to high (red) score. **(b)** Pearson’s correlation analysis preliminarily demonstrates that high-risk score (marked with *) is negatively correlated with some immune-related pathways (marked with **). Red indicates a positive correlation coefficient while green indicates a negative correlation. **(c-d)** Boxplots showing the score of immune-related cells **(c)** and functions **(d)**. Adjusted *p-*values are labelled as: ***, *p* < 0.001; **, *p* < 0.01; *, *p* < 0.05; ns, not significant

To elucidate the potential signaling pathways associated with the 10-FRG gene signature, the mRNA matrix of patients was split by risk score into low- and high-risk group and analysed with ssGSEA. As shown in Fig. 9a, “p53 signaling pathway”, “cell cycle”, “DNA replication”, “mismatch repair” and “RNA degradation” were enriched in high-risk samples, while a variety of immune-related pathways including “B cell receptor signaling pathway”, “T cell receptor signaling pathway”, “acute myeloid leukaemia”, “FcγR mediated phagocytosis”, “FcεRI signaling pathway”, and “primary immunodeficiency” were enriched in the low-risk group. Moreover, Pearson’s correlation analysis showed that the risk score exhibited a positive correlation with most of these pathways(Fig. 9b). Therefore, these results preliminarily indicated that the 10-FRG signature may mainly affect immune-related and cancer-related signaling pathways, and dysregulation of these pathways may be associated with tumour development and progression.

### Immune cell infiltration pattern between the two risk groups

Generally, the pattern of infiltrating immune cells in the tumour microenvironment (TME) is distributed diversely between subgroups. As illustrated in Fig. 9c and d, aDCs, B cells, iDCs, mast cells, pDCs, the type II IFN response and T cell co-stimulation which are involved in the antigen presentation process were more highly activated in low-risk samples (*p* < 0.05), especially iDCs and B cells (*p* < 0.001). The results also showed that immune effector cells including CD8+ T cells, NK cells and tumour infiltrating lymphocytes (TIL) were downregulated in the high-risk group, and the scores of T helper cells, Th2 cells and regulatory T cell Treg presented similar results. The aforementioned results indicated that a high FRG-related risk score may be relevant to an immunosuppressed status.

## Discussion

An increasing number of studies have confirmed that ferroptosis plays a crucial role in carcinogenesis and oncotherapy [11, 25]. The construction of prognostic models based on public databases and next generation sequencing (NSG) provides more comprehensive clinical-genetic prognostic value and prognostic models based on ferroptosis are becoming a research hotspot for predicting OS in different cancers [22–24, 26, 27].However, the predictive value of ferroptosis-related genes for the OS of OSCC patients’ remains unknown and warrants further study. In this study, we comprehensively analysed the 127 ferroptosis-related genes expression patterns and their correlation with prognosis in OSCC. A novel prognostic signature based on 10 ferroptosis- related genes was first constructed and a nomogram to predict OS for patients with OSCC was subsequently established. ssGSEA finally revealed some cancer-related and immune-related pathways that affect the prognosis of OSCC. These results demonstrated that the novel FRG signature has the potential to accurately predict OSCC prognosis and could provide some insights into the development of novel ferroptotic biomarkers and targeted therapies.

In this study, more than half of the genes were expressed differentially between OSCC and normal samples. GO and KEGG analysis suggested that these 86 DE-FRGs were mainly enriched in principal ferroptosis-related functions and pathways, such as “response to metal ion”, “response to oxidative stress”, “iron ion binding”, “glutathione metabolism and fatty acid metabolism”. These results preliminarily suggested that ferroptosis-related genes and pathways might be involved in the development of OSCC and could be used to develop a prognostic signature.

Hence, 10 FRGs including ATG5, BID, ACO1, GOT1, AKR1C3, GLS2, ALOX15, SCO2, MAP1LC3A and MAP3K5, were determined to be the most powerful prognostic biomarkers based on minimum criteria with LASSO analysis. Ferroptosis is usually accompanied by excessive iron accumulation, lipid peroxidation, glutamate and antioxidant metabolism [9, 10]. In this regard, these genes can fall into four categories [28]: (1) ATG5, MAP1LC3A, and ACO1 are related to iron metabolism. ATG5 and MAP1LC3A activate autophagy through the ATG5-ATG7-NCOA4 pathway, leading to ferritin degradation and thereby intracellular unstable iron accumulation, ultimately promoting ferroptosis in fibroblasts and cancer cells [29]. ACO1 is an iron sensor that functions as an aconitase to convert citrate to isocitrate and then control iron levels and ferroptosis [30]; (2) MAP3K5, ALOX15, and AKR1C3 regulate lipid metabolism. In details, MAP3K5, also known as ASK1, is involved in the ASK1-p38 axis and activated by lipid ROS accumulation, implementing ferroptosis in lung cancer cells [31]. Inhibition of ALOX15 contributes to abrogation of lipid peroxides accumulation; thus, ferroptosis resistant melanoma cells efficiently activate NRF2 to elevate the level of AKR1C3 and lead to a negative regulation of ALOX15 [32]. (3) GLS2, GOT1 and BID are required for energy metabolism (glutamate metabolism). Glutamine (Gln) is first deamidated in mitochondria to glutamate (Glu) by glutaminase GLS, following conversion to a-KG by GOT1. Elevation of GLS2 and GOT1 increases the level of a-KG and reduces cysteine import. Both cysteine limitation and glutaminolysis increase ROS content and sensitize melanoma cells to chemically-induced ferroptosis [33]. In addition, BID transactivation and the subsequent mitochondrial fission, ROS accumulation and loss of mitochondrial membrane potential link ferroptosis to oxytosis pathway signaling in neuronal cells [34]; (4) SCO2 is important to (anti-)oxidant metabolism. SCO2 plays a putative ROS defensive role in human cell lines and double deletion of SCO2 and SOD1 incaeases ROS, resulting from superior sensitivity to oxidative stress [35]. Although some of these genes have been shown to be involved in the development and progression of OSCC, to know whether these genes influence the survival of OSCC patients by regulating ferroptosis need more clinical and basic research.

The gene signature classified patients into two groups and K-M curve analysis showed that patients with high-risk scores had a significantly worse prognosis. Univariate and multivariate Cox analysis confirmed the FRG-based risk score could act as an independent predictor for OS. However, none of the clinical factors were significantly related to the risk score. This was different from the prognostic model for OSCC based on another gene set [36]. However, there was a tendency for a higher FRG-based risk score to be associated with a more advanced clinical stage.

The nomogram can combine genetic and clinical information easily to calculate and predict a personalized survival rate of cancer patient, which has great value in helping doctors make decisions regarding diagnosis and treatment. Therefore, we developed an FRG signature-based nomogram and confirmed its good accuracy and sensitivity in validation sets. These results indicated that the nomogram was a favourable and reliable model for prognosis prediction in OSCC patients and showed great application potential.

It was demonstrated that the most vital ways in which ferroptotic cancer cells interact with the antitumour immunity were phagocytosis, migration, maturation, antigen processing, and cross-presentation by dendritic cells [37]. To determine whether the poor prognosis in high-risk patients was related to tumour cell-mediated immunity, ssGSEA between the different risk groups was performed. The DEGs between subgroups were enriched in a variety of cancer and immune-related pathways. Additionally, the risk score showed a negative correlation with a series of immune-related pathways in the Pearson’s correlation analysis. The aforementioned results implied that the ferroptosis-related gene signature may connected with the dysregulation of cancer-related and immune-related pathways.

Furthermore, additional quantitative research showed that the high-risk group contained a higher fraction of some immune-related cells and functions. According to previous studies, ferroptotic cancer cells can release damage-associated molecular patterns (DAMPs) or lipid mediators that have been found to attract antigen-presenting cells (APCs) to ferroptotic dying cells, subsequently, triggering a series of innate and adaptive immune responses [37, 38]. Consistent with a prior study [23], our study also indicated that antigen presentation process related cells and functions were significantly more activated in the low-risk group, especially iDCs and B cells. It should also be noted that iDCs and B cells favour antitumour activity in OSCC [39]. Moreover, the present results suggested that immune effector cells including CD8 + T cells, NK cells and TILs, were downregulated in the high-risk group. CD8 + T cells have been shown to induce lipid peroxidation in cancer cells and sensitize cells to ferroptosis via IFNγ [40]. A higher density of these immune cells was found in tertiary lymphoid structure-positive patients and contributed to a better 5-year overall survival for OSCC [41]. Th2 cells and Tregs play a notable role in tumour immune evasion. Poonam R et al. [42] reported that Th2 cells and Tregs were both associated with OSCC progression, and their upregulation increased the risk of death. These aforementioned studies of Th2 cells and Tregs were inconformity with our results. Therefore, the exact role of these immune cells in ferroptosis and immune evasion needs further investigation. Consequently, these results preliminarily indicated that the worse prognosis in the high-risk group might result from the dysregulation of antitumour immunity and left open a further question: how does ferroptosis affect the development of OSCC through antitumour immunity.

Compared with previous studies, our study was the first to construct an FRG-based prognostic signature and nomogram for predicting prognosis in OSCC and preliminarily revealed the relationship between ferroptosis-related genes and immune status. However, there were still several limitations in our research: First, our gene prognostic models were constructed and validated with the TCGA cohort, and other available external databases and prospective data in reality will be required to evaluate its clinical predictive value in the future. Second, further studies need to perform basic experiments to explore the relationship between ferroptosis and our prognostic signatures. Finally, the underlying correlation between the risk score and antitumour immunity remains to be further investigated. Therefore, the conclusion obtained from this study needs more detailed verification in view of the above limitations.

## Conclusions

In conclusion, we used bioinformatics analysis to construct a new 10-FRG prognostic model with good predictive value that was capable of independently predicting the overall survival of OSCC patients. The nomogram model of risk factors and clinical parameters provides a new understanding of the prognostic value of ferroptosis-related genes in OSCC and new insight for future anticancer immunotherapies based on potential FRG targets. In our future study, the predictive value of the model needs to be verified by clinical data and the inherent mechanism of ferroptosis-related genes and antitomour immunity needs to be unveiled.

## Supplementary Information

**Additional file 1.**

## Data Availability

Detailed information of FRGs was summarized in Additional file 1: Table S1.The datasets generated and/or analyzed during the current study are available in the TCGA repository (https://portal.gdc.cancer.gov).

## Abbreviations

OSCC: Oral squamous cell carcinoma
FRG: Ferroptosis-related genes
OS: Overall survival
TCGA: The Cancer Genome Atlas
LASSO: Least absolute shrinkage and selection operator
ROC: Receiver operating characteristic
AUC: Area under the curve
HR: Hazard ratio
ssGSEA: Single-sample gene set enrichment analysis
DEGs: Differentially expressed genes
DE-FRGs: Differentially expressed ferroptosis-related genes
FDR: False discovery rate (FDR)
GO: Gene Ontology
KEGG: Kyoto Encyclopedia of Genes and Genomes
PPI: Protein-protein interaction
K-M: Kaplan-Meier
CI: Confidence interval
